# Association Analysis of Stem Rust Resistance in U.S. Winter Wheat

**DOI:** 10.1371/journal.pone.0103747

**Published:** 2014-07-29

**Authors:** Dadong Zhang, Robert L. Bowden, Jianming Yu, Brett F. Carver, Guihua Bai

**Affiliations:** 1 Department of Agronomy, Kansas State University, Manhattan, Kansas, United States of America; 2 USDA–ARS Hard Winter Wheat Genetics Research Unit, Manhattan, Kansas, United States of America; 3 Department of Agronomy, Iowa State University, Ames, Iowa, United States of America; 4 Plant Science Department, Oklahoma State University, Stillwater, Oklahoma, United States of America; Nanjing Agricultural University, China

## Abstract

Stem rust has become a renewed threat to global wheat production after the emergence and spread of race TTKSK (also known as Ug99) and related races from Africa. To elucidate U.S. winter wheat resistance genes to stem rust, association mapping was conducted using a panel of 137 lines from cooperative U.S. winter wheat nurseries from 2008 and simple sequence repeat (SSR) and sequence tagged site (STS) markers across the wheat genome. Seedling infection types were evaluated in a greenhouse experiment using six U.S. stem rust races (QFCSC, QTHJC, RCRSC, RKQQC, TPMKC and TTTTF) and TTKSK, and adult plant responses to bulked U.S. races were evaluated in a field experiment. A linearization algorithm was used to convert the qualitative Stakman scale seedling infection types for quantitative analysis. Association mapping successfully detected six known stem rust seedling resistance genes in U.S. winter wheat lines with frequencies: *Sr6* (12%), *Sr24* (9%), *Sr31* (15%), *Sr36* (9%), *Sr38* (19%), and *Sr1RS^Amigo^* (8%). Adult plant resistance gene *Sr2* was present in 4% of lines. *SrTmp* was postulated to be present in several hard winter wheat lines, but the frequency could not be accurately determined. *Sr38* was the most prevalent *Sr* gene in both hard and soft winter wheat and was the most effective *Sr* gene in the adult plant field test. Resistance to TTKSK was associated *with* nine markers on chromosome 2B that were in linkage disequilibrium and all of the resistance was attributed to the *Triticum timopheevii* chromosome segment carrying *Sr36*. Potential novel rust resistance alleles were associated with markers *Xwmc326-203* on 3BL, *Xgwm160-195* and *Xwmc313-225* on 4AL near *Sr7*, *Xgwm495-182* on 4BL, *Xwmc622-147* and *Xgwm624-146* on 4DL, and *Xgwm334-123* on 6AS near *Sr8*. *Xwmc326-203* was associated with adult plant resistance to bulked U.S. races and *Xgwm495-182* was associated with seedling resistance to TTKSK.

## Introduction

Stem rust (SR), caused by *Puccinia graminis* Pers.:Pers. f. sp. *tritici* Erikss. & E. Henn., historically was a destructive disease in wheat (*Triticum aestivum* L.) worldwide [1]. In the United States, SR occurred frequently from the 1920s to 1960s and caused yield losses up to 50% in severe epidemic years [2]. Since the late 1970s, major SR epidemics have not been reported due to the successful deployment of resistance genes in commercial wheat cultivars in conjunction with the eradication of common barberry (*Berberis vulgaris* L.) [2]. The emergence and spread of race TTKSK (also known as Ug99) and related races from Africa are of great concern because they have overcome many important resistance genes used in commercial production [1], [3]–[6]. Race TTTTF, first detected in the U.S in 2000, is also virulent on a large number of important resistance genes [7]. Achieving more durable resistance will depend on deploying diverse combinations of race-specific qualitative resistance and/or race-nonspecific quantitative resistance genes [8].

Numerous SR resistance genes have been identified, but many have limited usefulness in agriculture [9], [10]. Only six of the 30 genes listed from *T. aestivum* were effective against all tested races, and several of these conferred inadequate levels of resistance by themselves [9]. *Sr2* derived from *T. turgidum* is the only proven durable race-nonspecific SR resistance gene, although several newly identified adult plant resistance genes may eventually be shown to be durable [9], [11]. Other important resistance genes from alien species include *Sr24*, *Sr31, Sr1RS^Amigo^*, *Sr36* and *Sr38*. Although these genes have now been individually defeated by the new races [5], [6], [9], they are still useful in combinations. Other alien genes such as *Sr22, Sr25*, *Sr26*, *Sr35*, *Sr39*, *Sr40*, and *Sr44* remain effective against the new races but are not yet widely deployed due to concerns about linkage drag [9]. Alien chromosome segments are being shortened to reduce linkage drag for many sources of resistance [12], [13].

An essential step in developing and deploying genetic resistance resources in U.S. winter wheat breeding programs is to understand the existing complement of SR resistance genes. Early gene postulation work based on characteristic low infection types (ITs) against a range of stem rust cultures identified resistance genes *Sr5*, *Sr6*, *Sr7b*, *Sr8*a, *Sr9a, Sr10*, *Sr11*, *Sr12*, *Sr17*, *Sr36*, *SrMcN*, and *SrTmp* in some U.S. wheat cultivars [14]–[16]. Jin and Singh [17] postulated the presence of *Sr6*, *Sr24*, *Sr31*, *Sr36, Sr1RS^Amigo^* or *SrTmp* in a set of 37 hard and 19 soft winter wheat cultivars. Yu et al. [18] detected marker alleles associated with *Sr2*, *Sr24*, *Sr36* and *Sr1RS^Amigo^* in a set of 31 U.S. wheat germplasm lines. Olson et al. [19] detected markers for *Sr24*, *Sr31*, *Sr36* and *Sr1RS^Amigo^* in a collection of 776 U.S. cultivars. Although these reports have overlapping results, they are each incomplete representations of the full complement of resistance genes.

Association studies have been used to discover and validate both major genes and quantitative trait loci in different plant species. In wheat, association mapping was used to identify SR resistance genes in CIMMYT spring wheat germplasm [18], [20], [21] and Ethiopian durum wheat [22], [23] but using association mapping to study SR resistance in U.S. wheat has not been reported. This study analyzed a set of elite breeding lines from major U.S. winter wheat breeding programs using association mapping and a newly developed algorithm to convert complex Stakman IT scores [24] to a linear scale. Zhang et al. [25] previously described the genetic diversity, population structure, and linkage disequilibrium relationships in this population and it was successfully used for association mapping of resistance to *Soilborne wheat mosaic virus* [26]. Our objectives in this study were to: 1) evaluate the IT data conversion method for detecting stem rust resistance genes, 2) validate reliability of DNA markers linked to known SR resistance genes in U.S. winter wheat, 3) discover potential new genes and/or markers for wheat SR resistance, and 4) determine the composition and frequency of SR resistance genes in U.S. winter wheat elite breeding lines.

## Materials and Methods

### Plant materials

A set of 137 U.S. elite breeding lines and cultivars was selected from the 2008 USDA-ARS Southern (SRPN, n = 44) and Northern (NRPN, n = 28) Hard Winter Wheat Regional Performance Nurseries, and the USDA-ARS Uniform Eastern (UESRWWN, n = 34) and Southern Soft Red Winter Wheat Nurseries (USSRWWN, n = 31), after removal of sibling lines. These accessions included 72 hard winter wheat (HWW) and 65 soft winter wheat (SWW) lines (Table S1). Seed was provided by the breeding program at Oklahoma State University, Stillwater, OK.

### Marker data

Leaf tissue was sampled from a single plant at the two-leaf stage, and DNA was extracted using the cetyltrimethyl ammonium bromide (CTAB) method [25]. PCR amplifications were performed in a DNA Engine Thermal Cycler (Bio-Rad Laboratories, Hercules, CA) with 12 µl PCR mixture containing 1.2 µl of 10× PCR buffer (Bioline, Taunton, MA), 2.5 mM of MgCl_2_, 200 µM of each dNTP, 50 nM of forward tailed primer that was synthesized by adding 18 bp of M13 tail to 5′ end of each forward primer, 250 nM of reverse primer and 200 nM of M13 fluorescent-dye labeled primer, 0.6 U of *Taq* DNA polymerase, and about 80 ng template DNA. Specific PCR programs were used according to available published papers to amplify marker fragments for known genes; otherwise, a touchdown PCR program was used [25].

All accessions were genotyped for 289 markers (Table S2), including 272 SSRs distributed over all 21 chromosomes (http://wheat.pw.usda.gov/GG2/index.shtml) and 17 previously reported markers closely linked to SR resistance genes (Table 1). Wheat lines or cultivars with known genes were used as controls for identifying the correct PCR fragment sizes of each marker. PCR products were analyzed in an ABI DNA Analyzer (Applied Biosystems, Foster City, CA), and marker data were scored using GeneMarker version 1.6 (SoftGenetics LLC, State College, PA) and manually checked twice for accuracy. Alleles from each marker were recorded following Breseghello et al. [27]. Marker alleles were named by a combination of primer name and target fragment size (bp). For example, *Xscm9-241*is the marker allele for the 1B/1R translocation, where Xscm9 is the primer name and 241 is the fragment size in bp including the 18 bp M13 sequence. The number of alleles recorded for each primer is listed in Table S2. Fifty-eight percent of amplified SSR alleles were at less than 5% frequency and so were excluded from the association analysis. The number of remaining alleles for analysis was 1042.

**Table 1:** List of markers associated with rust resistance genes, assigned chromosome, and number of alleles detected for each marker across 137 U.S. wheat accessions.

Entry	Marker	Chr.	No. of alleles	Gene	Positive Control	References
**1**	*XcsSr2*	3BS	3	*Sr2*	Scout 66	[57]
**2**	*Xsr2stm559*	3BS	8	*Sr2*	Scout 66	[58]
**3**	*XSr2X3B028F08*	3BS	2	*Sr2*	Scout 66	[59]
**4**	*Xcfa2019*	7AL	6	*Sr22*	Sr22Tb	[60]
**5**	*XSr24#12*	3DL	2	*Sr24*	Jagalene	[61]
**6**	*XSr24#50*	3DL	2	*Sr24*	Jagalene	[61]
**7**	*Xbarc71*	3DL	7	*Sr24*	Jagalene	[61]
**8**	*XSr26#43*	6AL	0	*Sr26*		[61]
**9**	*Xscm9*	1B/1A^a^	2	*Sr31, Sr1RS^Amigo^*	Amigo	[45]
**10**	*Xgwm319*	2BS	3	*Sr36/Sr40*	Vista	[46]
**11**	*Xgwm374*	2BS	6	*Sr40*	RL6088	[62]
**12**	*Xwmc477*	2BS	6	*Sr36/Sr39/Sr40*	Vista	[46], [62]
**13**	*Xwmc474*	2BS	14	*Sr40*	RL6088	[62]
**14**	*Xventriup.Ln2*	2AS	2	*Sr38/Yr17/Lr37*	Madsen	[47]
**15**	*Xcfd43*	2DS	6	*Sr6*		[48]
**16**	*Xwmc453*	2DS	12	*Sr6*		[48]
**17**	*Xgwm484*	2DS	24	*Sr6*		[48]

a *Xscm9* acts as a rye-specific SSR marker with two fragments amplified, 225 bp and 241 bp.

A fragment of 225 bp (forward primer tailed) indicates the T1RS•1BL chromosome, and resistance gene *Sr31* and 241 bp indicates the T1RS•1AL chromosome and gene *Sr1RS^Amigo^*.

### Stem rust evaluation

All wheat accessions were evaluated for seedling resistance to races QFCSC, QTHJC, RCRSC, RKQQC, TPMKC, TTTTF and TTKSK in a greenhouse and for adult plant resistance to bulked U.S. races (QFCSC, QTHJC, RCRSC, RKQQC and TPMKC) in the field at the USDA Cereal Disease Laboratory in St. Paul, MN in 2008. Field disease ratings were based on the percentage infection of the stems using the modified Cobb scale [28] when susceptible controls reached 60–70% severity. Seedling IT was scored using the Stakman scale [24]. Details on plant culture, inoculation methods, and scoring methods for the greenhouse and field experiments were described [29].

To meet the data format required for association analysis, original seedling IT data were converted to a 0–9 linear disease scale as we described in a preliminary report [30]. Simple infection types were converted as follows: 0, 1−, 1, 1+, 2−, 2, 2+, 3−, 3 and 3+ were coded as 0, 1, 2, 3, 4, 5, 6, 7, 8 and 9, respectively. For lines with heterogeneous reactions, only the most prevalent IT was used. The semicolon symbol for hypersensitive fleck “;” was converted to 0. IT 4 was converted to 9. Special annotation code “S” for susceptible was converted to 9 and “S LIF” for low infection frequency was converted to 8. Special annotation codes “C” for extra chlorosis and “N” for extra necrosis were ignored. Double minus and double plus annotations were converted to single minus and single plus, respectively. Complex ranges such as ;12+ were first collapsed to ;2+. Then the first and last ITs of the range were converted and averaged with the first IT being double-weighted because the most prevalent IT is always listed first. Mesothetic reaction types X−, X, and X+ were converted to linearized scores of 4, 5, and 6, respectively. Y and Z mesothetic infection types were treated similarly to X. The conversion algorithm is implemented with examples as an editable Excel spreadsheet in Table S3. Each IT score was based on one replication comprising five to six seedlings per isolate, except for TTKSK, in which two replications were used for each accession and a mean value was used for association analysis.

### Gene postulation

Named stem rust resistance genes present in each accession were postulated by the presence of diagnostic markers from published reports (Table 1). Expected ITs [10], [14], [31] and virulence/avirulence relationships were subsequently compared to observed ITs. For the purpose of gene postulation, the lower IT was assumed to be correct when ITs were heterogeneous. When observed infection types were substantially higher than expected infection types, the postulated resistance gene was assumed to be absent. When the observed infection types were lower than expected infection types, presence of an additional unknown gene(s) was postulated and indicated by a “+”.

### Association analysis

Population structure (Q) was determined by STRUCTURE 2.2 [32] using 42 genome-specific markers across all arms of the 21 chromosomes. Six independent runs were conducted using the admixture model by assuming that individuals might have mixed ancestries. Then k, the number of subpopulations, ranging from 2 to 10 was evaluated using a burn-in length of 2×105 and run length of 1×105. The maximum likelihood of each k value, the variance among 10 runs and the pedigree information of each line were used to determine the optimal number of subpopulations. Individuals were assigned to a subpopulation if confidence estimated by the program was at least 0.5; if the confidence value was below 0.5, a combination of information on geographical origin, market class, and breeding history was considered to assign them to a reasonable subpopulation. Population structure information matrix Q (n×k), where n is the number of accessions assayed and k is the number of subpopulations defined, was used in association analyses.

The comparison among models was conducted following Yu et al. [33]. Pair-wise kinship (K) coefficients among the 137 accessions were estimated using two types of algorithms, ‘KL’ proposed by [34] and ‘KR’ suggested by [35], [36] using the program SPAGeDi ver. 1.2 [37]. For these two algorithms, KR gives more weight to rare alleles and provides more power to detect genetic structure, whereas KL does not suffer bias in the presence of low-frequency alleles but provides low power to detect structure.

TASSEL version 2.1 [38] was used for model selection based on the 271 markers, excluding the 18 previously reported markers linked to stem rust resistance genes. The EMMA algorithm [39] and ‘P3D’ [40] were set during the process, then the *P*-values observed from each model were aligned against the expected *P*-values. The expected *P*-values were calculated as *r*(*x_m_*)/271, where *r*(*x_m_*) is the rank of the *P*-value *x_m_* observed for the *m*th marker locus. A mean of the squared differences (MSD) between observed and expected *P*-values of all marker loci was calculated as a measure for the deviation of the observed *P*-values from the expected distribution. A high MSD value indicated a high rate of empirical type I error [41]. The model with the smallest MSD was used for final association analysis.

Association analysis was conducted using PROC MIXED in SAS (SAS Institute, Cary, NC). Marker alleles with a frequency lower than 5% were excluded for calculation. The threshold for claiming significance of associations was set to *P*<0.001. A distance-based cluster analysis was conducted using PowerMarker v. 3.25 [42] and the unweighted pair group method with arithmetic mean (UPGMA) based on Nei distance [43].

If a chromosome region had more than three significant markers associated with a given trait, linkage disequilibrium (LD) was evaluated according to the frequency of target alleles using TASSEL 2.1 (http://www.maizegenetics.net/tasselx) with 1000 permutations. Marker order and genetic distance between markers on a wheat chromosome were adopted from previously established consensus maps [44].

## Results

### Stem rust resistance of U.S. winter wheat

Significant variation in the responses to different rust races was observed among the U.S. wheat accessions. At the seedling stage, a relatively high proportion of the accessions was resistant (converted scale values of 0 to 4) to QFCSC (58.4%) and QTHJC (40.1%), with at least one-third of accessions showing flecks (converted scale 0). For races RCRSC, RKQQC and TPMKC, a lower proportion of accessions (27–37%) showed resistance, with 12–17% of accessions having flecks. About 53% and 64% accessions were highly susceptible (IT>3) to races TTKSK and TTTTF, respectively, and less than 10% of accessions showed flecks. In the field experiment, about 25% of accessions showed negligible symptoms of rust infection, and 62% of accessions showed 40% or lower SEV to U.S. bulked races in adult plants. A total of 28 accessions showed resistance to all races tested in both seedling and adult stages.

### Population structure and statistical model comparison

Structure analysis identified a high level of population structure in the association-mapping panel, and four was the optimal number of subpopulations, with three HWW and one SWW subpopulations. Further details about the population structure are listed by Zhang et al. [25]. The QK model had the smallest MSD values for all disease measurements in model tests, and thus provided the best control of the false positive rate among all models tested. Because QK^L^ had slightly smaller MSD value than QK^R^ for some measurements, it was selected for further association analysis.

### Detection of known stem rust resistance genes

All races used in this study are avirulent to *Sr24*. Marker allele *XSr24#50-212* for *Sr24* was associated with resistance to all races except TPMKC (Table 2). All 13 accessions carrying the allele were uniformly resistant or moderately resistant to the six U.S. races, including TPMKC, and TTKSK, except that ‘Wesley’ and possibly ‘TX03A0563’ appeared to be phenotypically heterogeneous for *Sr24* (Table 2). The marker for *Sr24* was present in each of the four subpopulations (Table S1).

**Table 2:** Markers associated with known stem rust resistance genes and newly detected loci, and mean rust ratings of accessions carrying these marker alleles after inoculation with seven stem rust races at the seedling stage and bulked U.S. races at the adult stage.

	Allele	Location	Resistance Gene^b^	Seedling IT^d^							Adult plant
				QFCSC	QTHJC	TTKSK	RCRSC	RKQQC	TPMKC	TTTTF	Severity, %
Marker for known *Sr* genes^a^	*Xscm9-241*	T1RS·1AL	*Sr1RS^Amigo^*	2.64	-	4.64	3.91	3.18	3.27	4.10	13.2
	*Xscm9-225*	T1RS·1BL	*Sr31*	2.14	1.75	-	2.81	3.85	2.39	3.10	13.0
	*Xventriup.Ln2*	2AS	*Sr38*	0.72	1.12	-	-	2.00	-	-	5.2
	*Xwmc477-176*	2BS	*Sr36*	0.64	1.20	1.73	-	-	-	-	15.5
	*Xcfd43-213*	2DS	*Sr6*	1.32	-	-	-	-	1.00	-	-
	*Xsr24#50-212*	3DL	*Sr24*	2.00	3.07	4.19	3.77	3.54	-	4.30	-
Newly detected marker associations	*Xbarc181-194*	1BL		-	-	6.14	-	-	-	-	-
	*Xgwm95-133*	2AS		-	0.25	-	2.38	-	-	3.13	-
	*Xwmc702-203*	2AS		-	2.00	-	-	-	3.28	-	12.6
	*Xwmc326-203*	3BL		-	-	-	-	-	-	-	16.4
	*Xgwm383-213*	3DL		-	-	-	2.38	-	-	-	-
	*Xgwm160-195*	4AL	*Sr7*	-	-	-	2.75	3.91	-	4.82	-
	*Xwmc313-225*	4AL	*Sr7*	-	-	-	3.40	-	-	-	-
	*Xgwm495-182*	4BL		-	-	4.25	-	-	-	-	-
	*Xwmc622-147*	4DL		-	-	-	2.86	3.57	-	2.43	-
	*Xgwm 624-146*	4DL		-	-	-	-	3.63	-	-	-
	*Xgwm540-143*	5BS		-	-	-	-	-	2.08	-	-
	*Xbarc 239-301*	5DL		-	-	4.47	-	-	-	-	-
	*Xgwm334-123*	6AS	*Sr8*	0.5	1.44	-	-	-	-	-	-
Negative alleles^c^	*Xgwm11-208*	1BS		-	-	-	8.18	8.24	7.19	8.76	50.3
	*Xwmc116-385*	7AL		-	-	-	7.43	7.79	7.21	-	-
	*Xbarc91-144*	2BS		6.13	6.40	8.67	-	-	-	-	50.0

a Means for seedling IT were calculated from the transformed 0 to 9 scale.

Only significant markers are shown (*P*<0.001).

b Postulated gene based on diagnostic marker or chromosome location.

c Not all negative alleles associated with susceptibility are listed.

d QFCSC (virulence/avirulence formula 5, 8a, 9a, 9d, 9g, 10, 17, 21, McN/6, 7b, 9b, 9e, 11, 24, 30, 31, 36, 38, Tmp, *Sr1RS^Amigo^*), QTHJC (5, 6, 8a, 9b, 9d, 9g, 10, 11, 17, 21, McN/7b, 9a, 9e, 24, 30, 31, 36, 38, Tmp, *Sr1RS^Amigo^*), RCRSC (5, 7b, 9a, 9b, 9d, 9g, 10, 17, 21, 36, McN/6, 8a, 9e, 11, 24, 30, 31, 38, Tmp, *Sr1RS^Amigo^*), RKQQC (5, 6, 7b, 8a, 9a, 9b, 9d, 9g, 21, 36, McN/9e, 10, 11, 17, 24, 30, 31, 38, Tmp, *Sr1RS^Amigo^*), TPMKC (5, 7b, 8a, 9a, 9d, 9e, 9g, 10, 11, 17, 21, 36, Tmp, McN/6, 9a, 9b, 24, 30, 31, 38, *Sr1RS^Amigo^*), TTTTF (5, 6, 7b, 8a, 9a, 9b, 9d, 9e, 9g, 10, 11, 17, 21, 30, 36, 38, Tmp, McN/24, 31, *Sr1RS^Amigo^*), and TTKSK (5, 6, 7b, 8a, 9a, 9b, 9d, 9e, 9g, 10, 11, 17, 21, 30, 31, 38, McN/24, 36, Tmp, *Sr1RS^Amigo^*).

Marker alleles *Xscm9-241* and *Xscm9-225* are diagnostic for *Sr1RS^Amigo^* that resides on wheat-rye translocation T1AL⋅1RS and for *Sr31* on translocation T1BL⋅IRS, respectively [45]. *Xscm9-241* was associated with resistance to all races except QTHJC (Table 2). Twelve accessions with *Sr1RS^Amigo^* showed resistance in both seedling and adult stages, but 5 accessions appeared to be phenotypically heterogeneous. *Xscm9-225* for *Sr31* was significantly associated with resistance to all races except TTKSK (Table 2). Seventeen accessions carrying *Xscm9-225* were uniformly resistant to all tested U.S. races and four appeared to be heterogeneous (Table S1). *Xscm9-225* was the marker with the lowest log_10_ *P*-value for seedling resistance to races RCRSC, TPMKC and TTTTF and for adult plant resistance.

Among the seven races tested, only QFCSC, QTHJC and TTKSK are avirulent to *Sr36*. Marker alleles *Xwmc477-176* and *Xgwm319-182* on chromosome 2B were reported to be diagnostic for *Sr36* [46]. In this study, both markers had identical results and were significantly associated with resistance to the three races and bulked U.S. races in the field (Table 2). Twelve of 15 accessions that carried the positive alleles showed near immunity to the three races at the seedling stage and high resistance to bulked U.S. races (Table S1). Accession ‘G61505’ was positive for both markers but susceptible to all races, indicating that *Sr36* was absent. ‘GA991209-6E33’ and ‘India Exp.’ carried the marker for *Sr31* in addition to the two markers for *Sr36* and were resistant to most races, but not to TTKSK, suggesting the absence of a functional *Sr36*. Thus, the two markers for *Sr36* produced false positives in the three accessions. All of the accessions with *Sr36* marker alleles for resistance were SWW.

Races QFCSC, QTHJC, RCRSC, RKQQC, and TPMKC are avirulent, and the others are virulent on *Sr38*. *Xventriup.ln2* is a marker linked to the *Sr38*/*Lr37*/*Yr17* gene cluster [47]. The marker was associated with resistance to QFCSC, QTHJC and RKQQC (Table 2). It was also associated with resistance to bulked isolates in the field. Twenty-four accessions exhibited uniform resistance, and two appeared to be heterogeneous for *Sr38* (Table S1).

Races QFCSC, RCRSC, and TPMKC are avirulent, and the others are virulent on *Sr6*. *Xcfd43-213* and *Xwmc453-130* linked to *Sr6* [48] showed significant association with resistance to QFCSC and TPMKC (Table 2). Twenty accessions had positive alleles for *Sr6*, and most showed high resistance to QFCSC and TPMKC; however, two accessions appeared to be heterogeneous, and three appeared to lack the phenotype for *Sr6*.

Marker *csSr2* for *Sr2* did not show significant association with resistance to any races in the association analysis, but it was detected in CO02W237, ‘Snowmass’, ‘Thunder CL’, ‘Tiger’ and ‘Scout 66’. *Sr2* was present in two HWW subpopulations. Scout 66, an old HWW cultivar from Nebraska, is known to possess *Sr2* [10]. Tiger from Kansas State University and the other three from Colorado State University are newer HWW cultivars/lines. Two other markers for *Sr2* were less diagnostic than *csSr2*, and thus also not significant for any races tested.

### Novel marker associations with resistance to TTKSK

**Figure 1:**
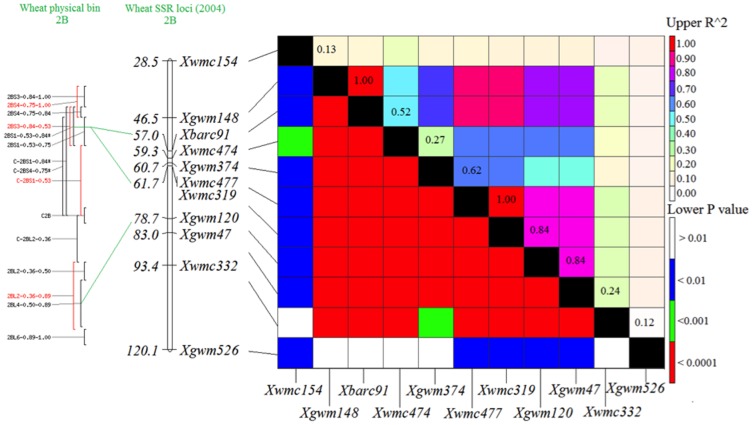
Physical bin map, linkage map, and linkage disequilibrium (LD) among 11 markers associated with resistance to race TTKSK on chromosome 2B. The upper diagonal indicates the LD level between markers reflected by *R^2^*, and the bottom diagonal indicates the statistical significance of LD between markers as reflected by *P*-values.

Seven marker alleles (*Xgwm148-127*, *Xbarc91-null*, *Xwmc474-141*, *Xgwm374-193*, *Xgwm120-null*, *Xgwm47-163* and *Xwmc332-165*) on chromosome 2B were associated with seedling resistance to TTKSK, QFCSC and QTHJC. These seven closely linked markers showed significant linkage disequilibrium (LD) with diagnostic markers *Xwmc477-176* and *Xgwm319-182* for *Sr36* (Fig. 1). In most cases, the seven linked markers identified the same positive lines as the markers for *Sr36* (Table 3).

**Table 3:** Phenotypes and haplotypes of accessions that carry positive alleles on chromosome 2B associated with resistance to TTKSK at the seedling stage.

Accession	Class	Gene postulation^1^	TTKSK-1	TTKSK-2	QFCSC	QTHJC	Xgwm148-127	Xbarc91-Null	Xwmc474-141	Xgwm374-193	Xwmc477-176	Xgwm319-182	Xgwm120-Null	Xgwm47-163	Xwmc332-165
G69202	SRW	Sr36, +	0	0	0;	0	+	+	+	+	+	+	+	+	+
P03112A1-7-14	SRW	Sr36, Sr38	0	0	0	0	+	+	+	−	+	+	+	+	+
INW0411	SRW	Sr31, Sr36$	0	0/;2	0	0	+	+	+	−	+	+	+	+	+
VA02W-555	SRW	Sr31, Sr36	0	0	0	0	+	+	+	−	+	+	+	+	+
VA04W-259	SRW	Sr36	0	0;	0	0	+	+	+	+	+	+	+	+	+
NC03-6228	SRW	Sr31$, Sr36	0	0	0	0	+	+	+	+	+	+	+	+	+
AR96077-7-2	SRW	Sr36	0	;1+	0	0	+	+	+	+	+	+	+	+	+
G61505	SRW	Sr36#	S	S	S	S	+	+	+	+	+	+	+	+	+
P04287A1-10	SRW	Sr36, +	2;	0;	0	0	+	+	−	−	+	+	+	+	+
G41732	SRW	Sr36, +	0	0	0	0/S	+	+	−	+	+	+	+	+	+
India Exp.	SRW	Sr31$, Sr36#	S	S	S/2	S/2-	+	+	−	+	+	+	+	+	+
NC04-15533	SRW	Sr36	0	0;	0;/;1	0	+	+	−	+	+	+	+	+	+
B030543	SRW	Sr36$	0	0/S	;	;	+	+	−	+	+	+	−	−	−
MD99W483-06-9	SRW	Sr31, Sr36	0	0	0	0	−	−	+	−	+	+	+	+	+
GA991209-6E33	SRW	Sr31, Sr36#, +	2+	2+ LIF	0	0	−	−	−	−	+	+	−	−	−

1 “+” denotes extra resistance that could not be attributed to postulated genes in the lines for at least one of seven races; “#”denotes putative nonfunctional alleles where phenotypes did not confirm gene postulations based on diagnostic markers; and “$” denotes that the functional allele appeared to be heterogeneous.

“S” denotes susceptible infection type (IT) 3 or 4; “/” denotes heterogeneous, the predominant type given first; “LIF” denotes low infection frequency with fewer pustules.

Marker allele *Xbarc181-194* on 1B was significantly associated with resistance to TTKSK and occurred only in the HWW accessions (Table S1). Among 13 accessions carrying this allele, three were susceptible to TTKSK; two had missing or contradictory phenotypic data; five lines also carry *Sr1RS^Amigo^* and the remaining three, CO03W043, CO03W139 and CO03064, had an IT of 2 to 2++ for TTKSK without carrying any known resistance gene.

Marker allele *Xgwm495-182* on 4BL was significantly associated with resistance to TTKSK (Table 2, Table S1). Five of the eight accessions carrying *Xgwm495-182* had no known gene for resistance to TTKSK. Two lines had *Sr36* and one had both *Sr24* and *Sr1RS^Amigo^*.

*Xbarc239-301* on 5DL was significantly associated with resistance to TTKSK, but nine of 15 positive accessions carried one or two other effective genes (Table S1). Three positive accessions were susceptible to all races, and two were susceptible to TTKSK. The lack of consistent association with resistance suggests that this association is spurious.

### Novel marker associations with resistance to other races

*Xgwm334-123* on 6AS was present in nine SRW lines and was associated with unexplained resistance to QFCSC and QTHJC in six accessions (Table S1). *Xgwm160-195* on 4AL and *Xwmc622-147* on 4DL were associated with resistance to RCRSC, RKQQC and TTTTF, and accessions with *Xwmc622-147* showed the lowest mean IT for TTTTF compared with all other marker alleles detected for this race. *Xgwm624-146* was associated with resistance to RKQQC. *Xwmc313-225* is tightly linked to *Xgwm160-195* and was also associated with resistance to RCRSC. *Xwmc702-203* on 2AS was associated with resistance to QTHJC and TPMKC. Eight accessions carrying *Xgwm95-133* on chromosome 2AS were resistant to QTHJC, RCRSC and TTTTF, and this marker appeared to be linked to the allele with the highest level of resistance to QTHJC (IT ‘0;’). *Xwmc326-203* on 3BL was only associated with adult plant resistance in the field.

Several other markers showed significant association with resistance, but most of the accessions that carry these markers also carry other known resistance genes. For example, eight accessions with the *Xgwm383-213* marker allele on chromosome 3DL had the lowest average IT (‘0;’ to ‘2’) to RCRSC, but six of them also carried either *Sr31* or *Sr24* (Table S1). Fourteen accessions carry *Xgwm540-143* on chromosome 5B, and seven of them had the markers for *Sr6*.

Several significant marker alleles were associated with high rust susceptibility (Table 2). For example, accessions with *Xwmc116-385* on chromosome 7A had an average IT higher than ‘3;’ for RCRSC, RKQQC and TPMKC. Six accessions with *Xgwm11-208* allele on chromosome 1B showed high susceptibility to RCRSC, TTTTF, TPMKC and RKQQC in seedling and adult stages.

## Discussion

Association mapping using the seedling IT linearization method described here (Table S3) successfully detected *Sr6, Sr24, Sr31, Sr36, Sr38*, and *Sr1RS^Amigo^* in U.S. winter wheat lines (Table 2). Utilization of seven isolates with known race specificities and previously published markers for these resistance genes allowed estimation of error rates for 42 marker-phenotype associations. For 11 instances where positive marker associations were not expected to occur because races were virulent on the particular resistance gene, none were significantly associated with resistance. For 31 instances where a positive marker association was expected because races were avirulent on the resistance genes, 26 associations were significant. Although the number of tests was relatively small, the results demonstrate the utility of association mapping with linearized ITs. Letta et al. [22] also used our IT linearization algorithm and similar association analyses to successfully map stem rust seedling resistance in durum wheat. The Stakman IT scale [24] is very useful for precise qualitative descriptions of rust resistance phenotypes and is routinely used to score rust reactions of experimental lines and characterize specific resistance genes. However, the system allows nonlinear or compound ITs such as X+, ;1N, or 13- that are not amenable to quantitative analysis. The linearization algorithm allows qualitative IT data to be converted for quantitative analysis.

Expected ITs based on marker genotypes were compared with actual resistance phenotypes to assess the prediction reliability of the markers. After accounting for heterogeneity of some wheat lines, the genotypic and phenotypic data showed excellent agreement for *Sr24*, *Sr31*, *Sr38*, and *Sr1RS^Amigo^* (Table S1). Each of these genes is on a non-recombining alien chromosome segment and markers were confirmed to be diagnostic in this U.S. winter wheat panel.

Marker *Xwmc477* was reported to be completely linked with *Sr36* on chromosome arm 2BS in two populations [46]. *Xgwm319* was also tightly linked at 0.9 cM distant in one population and completely linked in the other. In our study, marker alleles *Xwmc477-176* and *Xgwm319-182* for *Sr36* were positive for 15 lines. However, ITs indicated that *Sr36* was not present in 3 of the 15 lines (Table S1). Association mapping identified seven additional markers on 2B that were in linkage disequilibrium with markers for *Sr36* (Fig. 1). Based on similar ITs and race specificity (Table S1) and similar haplotypes among the lines (Table 3), the alien chromosome segment from *Triticum timopheevii* carrying *Sr36* was sufficient to explain the resistance associations on 2B. Susceptible lines ‘G61505’ and ‘India Exp.’ carried six or seven positive alleles from this LD block, in addition to *Xwmc477-176* and *Xgwm319-182*. Therefore, the *Sr36* haplotype appears to be intact and loss of *Sr36* gene function is likely. In contrast, susceptible line ‘GA991209-6E33’ had the negatively associated alleles at seven markers, in addition to positive alleles at *Xwmc477-176* and *Xgwm319*-182. This suggests that the alien translocation segment was disrupted in this line. Tsilo et al. [46] found a susceptible line, ‘CK9877’, that showed a negative allele for *Xwmc477*, but positive for *Xgwm319*. Olson et al. [19] reported that the positive allele of *Xwmc477* was associated with resistance in 54 of 57 cases. They attributed the remaining three susceptible lines to recombination or heterogeneity of the seed sources. *Xwmc477* appears to be the best marker for *Sr36*, but it appears not to be completely diagnostic.

Markers *Xcfd43* and *Xwmc453* for *Sr6* were tightly linked and diagnostic for *Sr6* in a diverse set of 46 wheat lines [48]. In the present study, marker alleles *Xcfd43-213* and *Xwmc453-130* were both positive for 20 accessions, but ITs indicated that *Sr6* was not present in 3 of the 20 lines, including ‘NE05430’, ‘NE05496’, and ‘Trego’. *Sr6* is on chromosome arm 2DS from common wheat and appears to have normal recombination rates [48]. The false positives are likely due to recombination between the resistance gene and the markers, which are 1.1 to 1.5 cM distant from *Sr6* [48].

Association mapping identified three potentially novel marker associations with resistance to race TTKSK for *Xbarc181-194* (1BL), *Xgwm495-182* (4BL), *Xbarc239-301* (5DL), and one with susceptibility to TTKSK for *Xbarc91-144* (Table 1). Njau et al. [49] mapped a stem rust resistance QTL in ‘Pavon 76’ to 1BL and inferred that it was the pleiotropic APR gene *Lr46*/*Yr29*/*Pm39*. However, that QTL was centered on *Xbarc80*, which is more than 50 cM distal from *Xbarc181*. Letta et al. [22] used association mapping to locate a stem rust resistance QTL near *Xbarc8* on 1BL in durum and suggested it was likely *Sr14*. However, *Xbarc8* is more than 10 cM proximal to *Xbarc181* and *Sr14* is not deployed in hexaploid wheat [10]. All 13 positive lines for *Xbarc181-194* were HWW and many were related to ‘TAM 107’ and/or Colorado experimental lines. Four Colorado lines showed good resistance to TTKSK, but contained no known resistance gene based on marker genotypes. They were previously postulated to carry *SrTmp* based on race specificity and infection phenotype (https://www.ars.usda.gov/SP2UserFiles/ad_hoc/54402000HardWinterWheatRegionalNurseryProgram/08SRPN.xls). Three of the four lines were positive for *Xbarc181-194*, which suggested that *Xbarc181-194* was associated with *SrTmp*. However, a gene thought to be *SrTmp* was recently mapped in the Colorado line ‘Ripper’ on 6DS [50]. It is therefore possible that the association with *Xbarc181-194* on 1BL is a spurious correlation between *SrTmp* and a marker that happens to be common in the same lines. In the present study, marker allele *Xgwm495-182* was associated with otherwise unexplained resistance to TTKSK in five lines. The magnitude of the effect of this locus appeared to be similar to *Sr24*, but it was not effective against other races (Table 2). Bhavani et al. [51] reported that a seedling stem rust resistance gene, temporarily designated *SrNing*, mapped near *Xgwm149* and *Xgwm495* on 4BL. *Xbarc239-301* on 5DL was also significantly associated with resistance to TTKSK, but most of the lines with *Xbarc239-301* carry either one or two known effective genes or are susceptible to TTKSK. Thus, the association with resistance is probably spurious. *Xbarc91-144* was associated with higher susceptibility to TTKSK. A null allele, *Xbarc91-null*, was part of the *Sr36* linkage block on 2BS (Fig. 1, Table 3). It is likely that *Xbarc91-144* detected the absence of *Sr36*. Therefore, the only novel association with seedling resistance to TTKSK that appears to be promising is *Xgwm495-182* on 4BL. Further work is needed to verify this marker association and possible relationship to *SrNing*.

Association mapping identified markers associated with resistance to races other than TTKSK on 2AS, 3BL, 3DL, 4AL, 4DL, 5BS, and 6AS, while markers were associated with susceptibility on 1BS and 7A (Table 2). *Xwmc702-203* and *Xgwm95-133* are tightly linked on 2AS and loosely linked to *Sr38*. Five of eight positive lines for *Xgwm95-133* and 10 of 19 positive lines for *Xwmc702-203* also carried *Sr38*, which may account for the association with resistance. However, *Xgwm95-133* was associated with resistance to TTTTF, which is virulent on *Sr38*. This suggests that *Xwmc702-203* and *Xgwm95-133* could be associated with a novel resistance gene on 2AS. Twenty-nine lines carried *Xwmc326-203* on 3BL, which was associated with resistance at the adult stage only. *Xwmc326-203* is distal on 3BL and is unlinked to the *Sr2* APR gene on 3BS. This marker was interesting because it was associated with stem rust APR in the winter wheat landrace variety, Kharkof. Markers *Xgwm160-195* and *Xwmc313-225* are tightly linked at the distal end of 4AL near *Sr7*. The race specificity is not consistent with allele *Sr7b* in the differential set, but the markers could be associated with a different allele of *Sr7*. *Xgwm383-213* on 3DL and *Xgwm540-143* on 5BS commonly occurred with other known genes and their effects are probably spurious. *Xwmc622-147*, which is 19 cM proximal to *Xgwm624-146* on 4DL, was associated with resistance to three different races. Both markers are distal to the pleiotropic locus *Lr67*/*Yr46*/*Sr55* on 4DL [52]. *Xwmc622-147* was interesting because it was associated with strong resistance to TTTTF. Marker allele *Xgwm334-123* was associated with resistance to two races. It is located at the tip of 6AS near *Sr8*. The race specificity was not consistent with *Sr8a*, so it could be associated with a different allele of *Sr8*. Marker *Xgwm11-208* on 1BS was associated with susceptibility and likely indicates the absence of *Sr31* and/or *Sr1RS^Amigo^*. Marker *Xwmc116-385* on distal 7AL was associated with higher susceptibility to three races, but the explanation is unclear. The most promising novel marker associations for races other than TTKSK are a possible APR gene near *Xwmc326-203* on 3BL, *Xgwm160-195* and *Xwmc313-225* on 4AL near *Sr7*, *Xwmc622-147* and *Xgwm624-146* on 4DL, and *Xgwm334-123* on 6AS near *Sr8*.

The impetus for this study was to assess the complement of stem rust resistance genes in U.S. winter wheat accessions from regional cooperative nurseries. Nineteen accessions (7%) were postulated to have no resistance genes for stem rust (Table S1). After correcting for false positives, frequencies of stem rust resistance genes in the U.S. winter wheat panel were *Sr2* (4%), *Sr6* (12%), *Sr24* (9%), *Sr31* (15%), *Sr36* (9%), *Sr38* (19%), and *Sr1RS^Amigo^* (8%). *Sr2* was found only in HWW and *Sr36* was found only in SWW. Fifty-two accessions (38%) were postulated to have some degree of additional unexplained resistance to one or more races. *SrTmp* was previously postulated to be present in four lines in the panel, but the frequency of *SrTmp* could not be determined because our associated marker was questionable. Association mapping yielded markers on 3BL, 4AL, 4BL, 4DL, and 6AS that may be associated with additional resistance genes, but all of them need to be validated.

Our results were in general agreement with Jin and Singh [17], Olson et al. [19], and Yu et al. [18]. The biggest difference was that *Sr38* from *Aegilops ventricosa* was found to be the most prevalent stem rust resistance gene in both hard and soft U.S. winter wheat. *Sr38* may have been overlooked previously because the phenotype is often confusing. Jin et al. [31] listed the IT of *Sr38* as ;23 and McIntosh et al. [10] listed the IT as X with larger pustules toward the leaf base [10]. The commonly used *Sr38* differential, ‘Trident’, often shows ITs of 0; to ;1, which suggests that it carries an additional gene. In the present study, lines putatively carrying only *Sr38* typically had seedling ITs of 0;, ;, ;1, ;13-, ;13, ;3, or 3; (Table S1). To account for the mesothetic reaction and pattern of larger pustules at the base, the typical IT for *Sr38* would best be scored as Z according to the Stakman scale. Fortunately, the marker *Xventriup.Ln2* appears to be very diagnostic for *Sr38*. *Sr38* does not provide protection against TTTTF or the TTKSK group of races, but it is effective against other North American races, especially at the adult stage. *Sr38* was the most effective SR gene in the field test. The average severity for lines carrying only *Sr38* was 5.2%, which is less than half of the value for the next most effective gene, *Sr31* (Table 2). *Sr38* is completely linked with other valuable traits like resistance to leaf rust (*Lr37*) and stripe rust (*Yr17*) that help to explain the prevalence of the segment in U.S. winter wheat breeding lines [47].

There were several reasons why some SR genes might have remained undetected in this study. First, the seven races used were all virulent on *Sr5*, *Sr9d*, *Sr9g*, *Sr21*, *SrMcN*, and all but one were virulent on *Sr10* and *Sr17*. Second, resistance alleles with a low frequency might be overlooked in this study because the power to detect an association is a function of allele frequency [53]. That might have affected the ability to detect a significant marker association for *Sr2*, which was present in only 4% of lines. Third, only one set of phenotypic data was available for adult plant field severity. This may have reduced the power to detect APR genes *Sr2* and *Lr34*/*Yr18*/*Sr57*, which is known to be present in HWW [54]. Fourth, the number of markers was insufficient for thorough genomic coverage. Although multiple alleles were recorded for most markers, 58% of amplified SSR alleles were at less than 5% frequency and so were excluded from the association analysis. Fifth, the size of the association mapping panel was relatively small. Nevertheless, known SR resistance genes and some potentially new resistance alleles were significantly associated with markers, thus demonstrating that archived qualitative rust infection type data can be linearized and then mined by association mapping.

This study analyzed archived stem rust phenotypic data from cooperative regional winter wheat trials from 2008. More than one half of the wheat accessions in the study were highly susceptible to race TTKSK, with only about 10% of accessions showing a high level of resistance with an IT of ‘0’ to ‘;’. Most of the effective resistance was attributable to *Sr24*, *Sr36*, and *Sr1RS^Amigo^*. In the intervening period, all of three of these genes have been defeated by new virulent races from Africa [5], [6], [55], so the risk of exotic races to U.S. winter wheat remains high. Efforts are underway to combine existing resistance genes with new stem rust genes such as *Sr22*, *Sr26*, and *Sr35* that are effective against the new races [56]. Race TTTTF, which is indigenous to the U.S., is also virulent on all but a few resistance genes. Although it has not yet become prevalent, improved resistance to TTTTF should also be a priority for winter wheat breeders in the U.S. [7].

## Supporting Information

Table S1 Linear alignment of neighbor-joining tree, rust rating data, significant positive markers, positive alleles (*P* < 0.001), and two most significant negative alleles.
(XLSX)

Table S2 List of 271 markers, assigned chromosome, and number of alleles detected across 174 U.S. wheat accessions.
(DOCX)

Table S3 Conversions of Stakman infection types to a linear scale.
(XLSX)
