# Supplementary material for: Association Analysis of Stem Rust Resistance in U.S. Winter Wheat
Source: PLoS One. 2014 Jul 29;9(7):e103747. doi: 10.1371/journal.pone.0103747 (PMC4114971; doi:10.1371/journal.pone.0103747)
Supplement: Table S2 — List of 271 markers, assigned chromosome, and number of alleles detected across 174 U.S. wheat accessions. (DOCX) [file pone.0103747.s002.docx]

**Table S2.** List of 271 markers, assigned chromosome, and number of alleles detected across 174 U.S. wheat accessions.

| **Marker** | **Chr.^a^** | **MAF^b^** | **NOA^c^** |  | **Marker** | **Chr.** | **MAF** | **NOA** |
| --- | --- | --- | --- | --- | --- | --- | --- | --- |
| *Xbarc158* | 1A | 0.7 | 4 |  | *Xbarc117* | 5A | 0.57 | 5 |
| *Xbarc83* | 1A | 0.62 | 6 |  | *Xbarc186* | 5A | 0.62 | 4 |
| *Xgwm357* | 1A | 0.45 | 6 |  | *Xbarc197* | 5A | 0.61 | 12 |
| *Xwmc24* | 1A | 0.35 | 12 |  | *Xbarc56* | 5A | 0.53 | 4 |
| *Xwmc59* | 1A | 0.36 | 10 |  | *Xgwm154* | 5A | 0.45 | 12 |
| *Xbarc181* | 1B | 0.6 | 8 |  | *Xgwm293* | 5A | 0.38 | 9 |
| *Xgdm36* | 1B | 0.89 | 4 |  | *Xgwm304* | 5A | 0.3 | 10 |
| *Xgwm11* | 1B | 0.33 | 10 |  | *Xgwm595* | 5A | 0.24 | 11 |
| *Xgwm18* | 1B | 0.36 | 8 |  | *Xwmc388* | 5A | 0.42 | 12 |
| *Xwmc419* | 1B | 0.25 | 11 |  | *Xwmc705* | 5A | 0.28 | 14 |
| *Xwmc44* | 1B | 0.2 | 20 |  | *Xcfd60* | 5B | 0.81 | 5 |
| *Xbarc169* | 1D | 0.61 | 6 |  | *Xgdm101* | 5B | 0.51 | 5 |
| *Xcfd63* | 1D | 0.41 | 13 |  | *Xgwm408* | 5B | 0.62 | 13 |
| *Xgwm147* | 1D | 0.69 | 5 |  | *Xgwm499* | 5B | 0.4 | 7 |
| *Xgwm337* | 1D | 0.23 | 12 |  | *Xgwm540* | 5B | 0.41 | 6 |
| *LR21214* | 1D | 0.57 | 5 |  | *Xwmc247* | 5B | 0.4 | 17 |
| *Xumn25* | 1D | 0.66 | 2 |  | *Xwmc363* | 5B | 0.91 | 4 |
| *Xumn26* | 1D | 0.66 | 2 |  | *Xwmc73* | 5B | 0.53 | 5 |
| *Xwmc147* | 1D | 0.34 | 7 |  | *Xcfd10* | 5D | 0.64 | 3 |
| *Xwmc216* | 1D | 0.62 | 7 |  | *Xgdm138* | 5D | 0.44 | 8 |
| *Xwmc222* | 1D | 0.82 | 6 |  | *Xgwm182* | 5D | 0.7 | 7 |
| *Xwmc609* | 1D | 0.52 | 9 |  | *Xgwm190* | 5D | 0.39 | 8 |
| *Xcfa2263* | 2A | 0.3 | 11 |  | *Xgwm212* | 5D | 0.63 | 6 |
| *Xgwm249* | 2A | 0.91 | 9 |  | *Xgwm292* | 5D | 0.34 | 11 |
| *Xgwm275* | 2A | 0.32 | 9 |  | *Xgwm358* | 5D | 0.8 | 6 |
| *Xgwm296* | 2A | 0.34 | 14 |  | *PINAD1* | 5D | 0.85 | 2 |
| *Xgwm356* | 2A | 0.2 | 19 |  | *Xwmc161* | 5D | 0.27 | 17 |
| *Xgwm95* | 2A | 0.52 | 6 |  | *Xwmc233* | 5D | 0.89 | 7 |
| *Xwmc177* | 2A | 0.31 | 14 |  | *Xgwm570* | 6A | 0.36 | 11 |
| *Xwmc181* | 2A | 0.53 | 8 |  | *Xgwm617* | 6A | 0.33 | 26 |
| *Xwmc296* | 2A | 0.52 | 9 |  | *Xwmc786* | 6A | 0.46 | 10 |
| *Xwmc522* | 2A | 0.19 | 20 |  | *Xbarc134* | 6B | 0.54 | 6 |
| *Xwmc644* | 2A | 0.2 | 26 |  | *Xgwm107* | 6B | 0.24 | 13 |
| *Xwmc819* | 2A | 0.26 | 22 |  | *Xgwm133* | 6B | 0.23 | 18 |
| *Xgwm120* | 2B | 0.23 | 12 |  | *Xgwm219* | 6B | 0.27 | 13 |
| *Xgwm148* | 2B | 0.32 | 11 |  | *Xgwm311* | 6B | 0.57 | 4 |
| *Xgwm47* | 2B | 0.43 | 12 |  | *Xgwm518* | 6B | 0.73 | 3 |
| *Xgwm526* | 2B | 0.5 | 13 |  | *Xgwm88* | 6B | 0.22 | 10 |
| *Xwmc154* | 2B | 0.38 | 10 |  | *Xwmc397* | 6B | 0.41 | 7 |
| *Xwmc332* | 2B | 0.34 | 13 |  | *Xwmc756* | 6B | 0.28 | 12 |
| *Xwmc361* | 2B | 0.63 | 5 |  | *Xbarc175* | 6D | 0.47 | 4 |
| *Xgdm35* | 2D | 0.34 | 16 |  | *Xbarc183* | 6D | 0.4 | 7 |
| *Xgwm102* | 2D | 0.35 | 24 |  | *Xbarc196* | 6D | 0.9 | 4 |
| *Xgwm157* | 2D | 0.83 | 5 |  | *Xbarc96* | 6D | 0.54 | 5 |
| *Xgwm210* | 2D | 0.56 | 3 |  | *Xcfd132* | 6D | 0.92 | 6 |
| *Xgwm261* | 2D | 0.29 | 10 |  | *Xcfd219* | 6D | 0.9 | 4 |
| *Xgwm320* | 2D | 0.28 | 8 |  | *Xcfd95* | 6D | 0.95 | 4 |
| *Xgwm539* | 2D | 0.23 | 17 |  | *Xbarc127* | 7A | 0.29 | 9 |
| *Xwmc41* | 2D | 0.73 | 3 |  | *Xbarc154* | 7A | 0.53 | 10 |
|  |  |  |  |  | *Xgwm130* | 7A | 0.34 | 11 |
| *Xbarc12* | 3A | 0.26 | 18 |  | *Xgwm276* | 7A | 0.43 | 24 |
| *Xbarc19* | 3A | 0.66 | 6 |  | *Xgwm60* | 7A | 0.54 | 11 |
| *Xbarc321* | 3A | 0.26 | 12 |  | *Xwmc116* | 7A | 0.32 | 11 |
| *Xbarc57* | 3A | 0.24 | 26 |  | *Xwmc283* | 7A | 0.25 | 15 |
| *Xcfa2134* | 3A | 0.19 | 21 |  | *Xwmc479* | 7A | 0.6 | 10 |
| *Xcfa2193* | 3A | 0.26 | 11 |  | *Xwmc488* | 7A | 0.37 | 12 |
| *Xgwm369* | 3A | 0.37 | 7 |  | *Xwmc83* | 7A | 0.41 | 10 |
| *Xgwm674* | 3A | 0.78 | 7 |  | *Xbarc72* | 7B | 0.76 | 4 |
| *Xwmc11* | 3A | 0.48 | 8 |  | *Xbarc85* | 7B | 0.98 | 3 |
| *Xwmc169* | 3A | 0.58 | 7 |  | *Xbarc94* | 7B | 0.22 | 11 |
| *Xwmc428* | 3A | 0.38 | 9 |  | *Xbarc95* | 7B | 0.46 | 5 |
| *Xwmc527* | 3A | 0.32 | 7 |  | *Xgwm46* | 7B | 0.32 | 12 |
| *Xwmc532* | 3A | 0.41 | 9 |  | *Xgwm537* | 7B | 0.27 | 17 |
| *Xbarc102* | 3B | 0.7 | 10 |  | *Xwmc323* | 7B | 0.7 | 7 |
| *Xbarc133* | 3B | 0.4 | 8 |  | *Xwmc364* | 7B | 0.5 | 6 |
| *Xbarc139* | 3B | 0.67 | 5 |  | *Xbarc111* | 7D | 0.95 | 6 |
| *Xbarc147* | 3B | 0.64 | 11 |  | *XbyagI* | 7D | 0.98 | 3 |
| *Xbarc164* | 3B | 0.6 | 10 |  | *Xgdm88* | 7D | 0.85 | 5 |
| *Xbarc344* | 3B | 0.23 | 18 |  | *Xgwm111* | 7D | 0.3 | 24 |
| *Xbarc77* | 3B | 0.22 | 12 |  | *Xgwm295* | 7D | 0.33 | 10 |
| *Xbarc84* | 3B | 0.69 | 3 |  | *XLR34CSL.VMS1* | 7D | 0.57 | 6 |
| *Xcfa2170* | 3B | 0.64 | 7 |  | *Xswm10* | 7D | 0.54 | 5 |
| *Xgwm108* | 3B | 0.3 | 7 |  | *Xwmc14* | 7D | 0.38 | 13 |
| *Xgwm114* | 3B | 0.7 | 13 |  | *Xwmc506* | 7D | 0.3 | 19 |
| *Xgwm156* | 3B | 0.32 | 14 |  | *Xwmc702* | 7D | 0.27 | 12 |
| *Xgwm181* | 3B | 0.2 | 16 |  | *Xbarc10* | UN | 0.42 | 13 |
| *Xgwm247* | 3B | 0.2 | 13 |  | *Xbarc123* | UN | 1 | 2 |
| *Xgwm285* | 3B | 0.33 | 12 |  | *Xbarc136* | UN | 0.92 | 4 |
| *Xgwm389* | 3B | 0.25 | 10 |  | *Xbarc145* | UN | 0.89 | 4 |
| *Xgwm493* | 3B | 0.5 | 10 |  | *Xbarc148* | UN | 0.38 | 10 |
| *Xgwm533* | 3B | 0.55 | 17 |  | *Xbarc173* | UN | 0.4 | 7 |
| *Xgwm547* | 3B | 0.7 | 4 |  | *Xbarc174* | UN | 0.66 | 9 |
| *Xgwm566* | 3B | 0.28 | 7 |  | *Xbarc180* | UN | 0.27 | 8 |
| *XXsts256* | 3B | 0.97 | 5 |  | *Xbarc195* | UN | 0.49 | 11 |
| *XXumn10* | 3B | 0.91 | 4 |  | *Xbarc206* | UN | 0.54 | 3 |
| *Xwmc231* | 3B | 0.41 | 8 |  | *Xbarc23* | UN | 0.78 | 10 |
| *Xwmc274* | 3B | 0.71 | 3 |  | *Xbarc239* | UN | 0.51 | 7 |
| *Xwmc291* | 3B | 0.55 | 9 |  | *Xbarc304* | UN | 0.39 | 9 |
| *Xwmc307* | 3B | 0.62 | 5 |  | *Xbarc352* | UN | 0.55 | 6 |
| *Xwmc326* | 3B | 0.21 | 17 |  | *Xbarc376* | UN | 0.28 | 15 |
| *Xwmc54* | 3B | 0.28 | 10 |  | *Xbarc68* | UN | 0.33 | 13 |
| *Xwmc754* | 3B | 0.37 | 16 |  | *Xbarc70* | UN | 0.35 | 6 |
| *Xwmc78* | 3B | 0.39 | 10 |  | *Xbarc76* | UN | 0.79 | 6 |
| *Xwmc808* | 3B | 0.29 | 10 |  | *Xbarc98* | UN | 0.4 | 6 |
| *Xcfd223* | 3D | 0.37 | 10 |  | *Xcfd193* | UN | 0.59 | 4 |
| *Xgdm72* | 3D | 0.37 | 7 |  | *Xcfd2* | UN | 0.83 | 3 |
| *Xgwm161* | 3D | 0.63 | 6 |  | *Xcfd58* | UN | 0.8 | 6 |
| *Xgwm3* | 3D | 0.45 | 8 |  | *Xcfd71* | UN | 0.29 | 13 |
| *Xgwm383* | 3D | 0.52 | 10 |  | *Xcfd86* | UN | 0.61 | 3 |
| *Xwmc549* | 3D | 0.8 | 4 |  | *Xgdm33* | UN | 0.42 | 13 |
| *Xbarc170* | 4A | 0.23 | 12 |  | *Xgwm10* | UN | 0.49 | 7 |
| *Xbarc78* | 4A | 0.42 | 10 |  | *Xgwm106* | UN | 0.84 | 5 |
| *Xcfa2256* | 4A | 0.75 | 6 |  | *Xgwm129* | UN | 0.63 | 4 |
| *DUP0108* | 4A | 0.57 | 6 |  | *Xgwm131* | UN | 0.29 | 14 |
| *Xgwm160* | 4A | 0.61 | 16 |  | *Xgwm191* | UN | 0.46 | 18 |
| *Xgwm397* | 4A | 0.62 | 9 |  | *Xgwm205* | UN | 0.36 | 8 |
| *Xgwm565* | 4A | 0.92 | 5 |  | *Xgwm213* | UN | 0.23 | 15 |
| *Xgwm610* | 4A | 0.43 | 7 |  | *Xgwm265* | UN | 0.9 | 6 |
| *Xwmc15* | 4A | 0.51 | 8 |  | *Xgwm319* | UN | 0.9 | 3 |
| *Xwmc313* | 4A | 0.35 | 9 |  | *Xgwm33* | UN | 0.37 | 12 |
| *Xwmc513* | 4A | 0.84 | 5 |  | *Xgwm4* | UN | 0.69 | 2 |
| *Xwmc650* | 4A | 0.19 | 11 |  | *Xgwm469* | UN | 0.39 | 22 |
| *Xwmc757* | 4A | 0.19 | 14 |  | *Xgwm515* | UN | 0.39 | 6 |
| *Xbarc1096* | 4B | 0.91 | 4 |  | *Xgwm614* | UN | 0.49 | 9 |
| *Xcfd39* | 4B | 0.36 | 20 |  | *Xgwm608* | UN | 0.36 | 20 |
| *Xgwm149* | 4B | 0.58 | 7 |  | *Xgwm644* | UN | 0.4 | 9 |
| *Xgwm495* | 4B | 0.52 | 7 |  | *Xgwm666* | UN | 0.8 | 4 |
| *Xwmc125* | 4B | 0.3 | 13 |  | *Xgwm67* | UN | 0.39 | 6 |
| *Xwmc349* | 4B | 0.36 | 7 |  | *Xgwm71* | UN | 0.33 | 13 |
| *Xwmc459* | 4B | 0.87 | 3 |  | *Xwmc149* | UN | 0.71 | 3 |
| *Xwmc47* | 4B | 0.72 | 4 |  | *Xwmc150* | UN | 0.44 | 21 |
| *Xwmc511* | 4B | 0.85 | 4 |  | *Xwmc173* | UN | 0.42 | 12 |
| *Xbarc91* | 4D | 0.29 | 14 |  | *Xwmc182* | UN | 0.36 | 6 |
| *Xcfd106* | 4D | 0.98 | 3 |  | *Xwmc206* | UN | 0.56 | 7 |
| *Xcfd23* | 4D | 0.62 | 10 |  | *Xwmc417* | UN | 0.34 | 8 |
| *Xgdm125* | 4D | 0.51 | 7 |  | *Xwmc48* | UN | 0.56 | 13 |
| *Xgwm194* | 4D | 0.27 | 9 |  | *Xwmc51* | UN | 0.93 | 3 |
| *Xgwm624* | 4D | 0.25 | 10 |  | *Xwmc56* | UN | 0.46 | 6 |
| *Xssr3A* | 4D | 0.2 | 16 |  | *Xwmc566* | UN | 0.59 | 3 |
| *Xssr3B* | 4D | 0.62 | 2 |  | *Xwmc631* | UN | 0.26 | 7 |
| *Xups4* | 4D | 0.3 | 9 |  | *Xwmc632* | UN | 0.2 | 18 |
| *Xwmc285* | 4D | 0.6 | 6 |  | *Xwmc773* | UN | 0.28 | 18 |
| *Xwmc331* | 4D | 0.54 | 7 |  | *Xwmc89* | UN | 0.51 | 16 |
| *Xwmc473* | 4D | 0.41 | 12 |  | *Xwmc93* | UN | 0.38 | 14 |
| *Xwmc52* | 4D | 0.44 | 8 |  | *WAXY4* | UN | 0.88 | 5 |
| *Xwmc622* | 4D | 0.26 | 15 |  | *WSMV1* | UN | 0.71 | 9 |

'UN' indicates no specific chromosome assigned.

^b^Major allele frequency

^c^Number of alleles identified from marker
